# Study on the Mechanism of Action of Baicalein in Inhibiting the Invasion of *Streptococcus agalactiae*

**DOI:** 10.3390/antiox15050544

**Published:** 2026-04-25

**Authors:** Lin Jiang, Xiaolei He, Yuxing Wang, Yang Liu, Xiubo Li, Fei Xu

**Affiliations:** Institute of Feed Research, Chinese Academy of Agricultural Sciences, No. 12 South Street, Haidian District, Beijing 100089, China

**Keywords:** *Streptococcus agalactiae*, invasion, virulence, baicalein, antioxidant

## Abstract

*Streptococcus agalactiae*, also known as *Group B Streptococcus* (GBS), is a major pathogen responsible for mastitis in dairy cows. It causes persistent and difficult-to-treat mammary infections, leading to reduced milk production. Baicalein, a flavonoid compound, exhibits anticancer, anti-inflammatory, and antibacterial activities; however, its specific mechanism of action against GBS remains unclear. This study aimed to investigate the mechanism by which baicalein inhibits GBS invasion of bovine mammary epithelial cells (bMECs). The results showed that baicalein at concentrations of 4 μg/mL or higher effectively inhibited 50% of the invasion of bMECs by GBS strain HB31 and exerted a concentration-dependent inhibitory effect on bacterial adhesion. The minimum inhibitory concentration (MIC) and minimum bactericidal concentration (MBC) of baicalein against HB31 were both greater than 1024 μg/mL. Therefore, the antibacterial effect of baicalein alone may not fully account for its mechanism; other pathways likely contribute to the reduced invasiveness of GBS. To elucidate the mechanism by which baicalein inhibits GBS invasiveness, this study investigated both bacterial metabolism and gene expression. Metabolomic analysis revealed that baicalein treatment led to the downregulation of amino acid metabolites, including alanine and aspartic acid, as well as nucleotide metabolites such as adenine and UMP in GBS HB31. Additionally, the NADH/NAD^+^ ratio increased while ATP levels decreased, indicating that the overall metabolic activity of GBS was suppressed. Transcriptomic analysis focused on changes in invasion-associated virulence genes. The results showed that the expression of *pbsP*, an invasion-associated virulence gene, was significantly reduced, while the expression of *hylB* and *cfb* showed downward trends that did not reach statistical significance. In contrast, the expression of *cylE* and the two-component system *vicKR* was upregulated. The upregulation of *cylE* may be related to baicalein-induced oxidative stress in HB31. Furthermore, HB31 suppressed *Nrf2*-*HO-1* mRNA expression, whereas baicalein activated the *Nrf2* signaling pathway and reduced HB31-induced *IL-6* and *NF-κB*mRNA expression. These findings provide new insights for the development of anti-virulence therapeutic strategies targeting GBS.

## 1. Introduction

*Streptococcus agalactiae*, also known as *Group B Streptococcus* (GBS), is an opportunistic Gram-positive bacterium capable of causing infections in humans [1], dairy cows [2], fish [3], and other animals, thereby leading to disease [4]. In clinical cases of GBS infection, high levels of bacteremia are frequently observed [5]. The bacterium can even cross the blood–brain barrier and cause meningitis, clearly demonstrating its pathogenic potential. GBS not only colonizes the mammary tissue of dairy cows for extended periods but also spreads among cows via milking equipment, leading to persistent and difficult-to-treat mastitis [6,7]. Damage to mammary epithelial cells and mammary tissue results in mastitis in dairy cows, which reduces milk production and causes significant economic losses [7]. Antibacterial therapy is widely used to treat GBS infections in both dairy herds and humans; however, clinically, antibiotic-resistant GBS strains are increasingly being identified [6,8]. The minimum inhibitory concentrations (MIC) of penicillin, ampicillin, and other β-lactam antibiotics against GBS isolates are gradually approaching their upper limits, rendering these antibiotics less effective [9]. Therefore, developing safe and effective alternatives to conventional antimicrobial drugs is imperative to maintain udder health in dairy cows.

Flavonoids are an important and diverse class of secondary plant metabolites and are currently a major focus of research due to their well-known anti-inflammatory and antibacterial properties [10]. Baicalein (5,6,7-trihydroxy-2-phenyl-4H-1-benzopyran-4-one) is a flavonoid compound derived from the roots of Scutellaria baicalensis and is commonly used for anticancer [11], and anti-inflammatory purposes [12,13,14]. Studies have shown that baicalein alleviates inflammation caused by Streptococcus suis by specifically inhibiting its hemolytic activity [14]. In addition, baicalein protects mice from Streptococcus pneumoniae-induced lung injury and death by modulating inflammatory cells and cytokines [15]. Baicalein also exhibits multiple mechanisms of action, including inhibiting biofilm formation, reducing toxin production, reversing or suppressing oxidative stress induced by antimicrobial resistance (AMR), and exerting synergistic effects [16]. Studies have shown that baicalein possesses anti-quorum sensing activity and biofilm-inhibiting potential against *Staphylococcus aureus, Aeromonas hydrophila* (AH), and *Candida albicans* [17,18]. In *S. aureus* and *Klebsiella pneumoniae*, baicalein alters the three-dimensional structure of the LuxS protein and downregulates luxS gene expression, thereby inhibiting the LuxS/AI-2 quorum sensing system and disrupting QS-regulated signaling pathways [19,20]. Furthermore, in mixed biofilms of *Streptococcus mutans* and *Candida albicans*, baicalein suppresses the expression of the adhesion factors *gtfB*, *gtfC*, and *gtfD* [21].Baicalein increases reactive oxygen species (ROS) levels in Acinetobacter baumannii and Staphylococcus aureus, inducing lethal oxidative stress and leading to bacterial death [22]. In addition to activating oxidative stress in bacteria, baicalein alleviates host oxidative stress caused by bacterial infection. Specifically, baicalein scavenges ROS and reduces oxidative stress injury in the liver [23]. In grass carp liver tissue, baicalein significantly upregulates the expression of superoxide dismutase (*SOD) and Catalase(CAT)* genes, which are downregulated under AH stress, thereby enhancing the antioxidant defense system and relieving oxidative stress [18,24]. Moreover, baicalein effectively attenuates H_2_O_2_-induced oxidative stress and subsequent apoptosis in cardiomyocytes [25]. In a rat model of irritable bowel syndrome, baicalein markedly ameliorates the elevation of malondialdehyde (MDA) levels and the inhibition of SOD activity, thus reducing oxidative stress in rat intestinal tissue [26]. Collectively, baicalein not only inhibits bacterial metabolism, quorum sensing, and virulence but also protects the host against oxidative stress and inflammatory injury.

These findings indicate that baicalein alleviates the host inflammatory response induced by Streptococcus pneumoniae, suggesting its potential as a therapeutic agent against *S. suis* and *S. pneumoniae*. However, its effect on GBS remains unclear. Therefore, in this study, adhesion and invasion assays were performed using GBS in the presence of baicalein. After incubating GBS with baicalein, metabolomic and transcriptomic analyses were conducted to explore the mechanism by which baicalein inhibits GBS invasion, thereby laying the foundation for its development as a therapeutic agent against GBS.

## 2. Materials and Methods

### 2.1. GBS Strains and Culture Conditions

GBS strain HB31 is a serotype III clinical isolate obtained from a cattle farm in Hebei Province, China. It was isolated from a milk sample collected from a cow with mastitis. Baicalein was purchased from Aladdin (CAS: 491-67-8). Bovine mammary epithelial cells (bMECs) were provided by Beijing Agricultural College. bMECs were cultured in DMEM high glucose medium (Gibco, Thermo Fisher Scientific, Waltham, MA, USA) supplemented with 10% fetal bovine serum (FBS; Gibco, Thermo Fisher Scientific, Waltham, MA, USA) and 1% penicillin/streptomycin (100 U/mL penicillin and 100 μg/mL streptomycin; Gibco, Thermo Fisher Scientific, Waltham, MA, USA). The cells were maintained in a humidified incubator at 37 °C with 5% CO_2_. The culture medium was renewed every 2–3 days. When the cell density reached 80–90% confluence, cells were washed twice with phosphate-buffered saline (PBS; Beijing Solarbio Science & Technology Co., Ltd., Beijing, China) and detached using 0.25% trypsin-EDTA (Beijing Solarbio Science & Technology Co., Ltd., Beijing, China) for passaging or subsequent experiments. The strain was cultured on 5% fetal bovine serum (FBS) agar or in LB broth supplemented with 5% FBS at 37 °C. LB broth was purchased from Qingdao Hi-tech Industrial Park Hope Bio-technology Co., Ltd. (Qingdao, China) Bacteria were grown to mid-log phase at 37 °C in a 5% CO_2_ atmosphere, and growth was monitored by measuring the optical density at 600 nm (OD_600_).

### 2.2. Determination of MIC and MBC for GBS

Twelve sterile test tubes were prepared, each containing 1 mL of sterile LB broth. Baicalein was serially diluted two-fold as follows: 1 mL of baicalein was added to the first tube and mixed thoroughly by pipetting up and down. Then, 1 mL of the mixture was transferred to the next tube, and the procedure was repeated sequentially until the last tube. This yielded baicalein concentrations of 1024, 512, 256, 128, 64, 32, 16, 8, 4, 2, 1, and 0.5 μg/mL. Subsequently, 1 mL of the prepared bacterial suspension (approximately 1 × 10^6^ CFU/mL) from each of the three strains was added to the 12 tubes. Three additional tubes containing only LB broth and bacterial suspension were prepared as positive controls, and one tube containing only LB broth served as a negative control. Three parallel replicates were set up for the above experiments. Simultaneously, a blank control group was included. All tubes were placed in a shaking incubator at 37 °C with constant agitation at 220 rpm for 24 h. After incubation, the lowest concentration of baicalein in the ascending concentration series at which the first clear tube (no visible bacterial growth) was observed was defined as the MIC. The OD_600_ of the samples was then measured to obtain corresponding values. From the well showing no visible bacterial growth, 50 μL of the culture was streaked onto a 5% FBS agar plate and incubated at 37 °C for 24 h. The lowest concentration of baicalein at which no colonies formed on the FBS agar plate was defined as the minimum bactericidal concentration (MBC).

### 2.3. GBS Invasion and Adhesion Assays

#### 2.3.1. Assay of Baicalein’s Inhibitory Effect on GBS Invasion

Invasion test was conducted as described by Salaheen et al. [27], with minor revisions. Bovine mammary epithelial cells (bMECs) were cultured in DMEM supplemented with 10% FBS and 1% streptomycin/penicillin (or streptomycin/streptozotocin, as specified). The cells were maintained in an incubator at 37 °C with 5% CO_2_ and were seeded into 24-well plates at a density of 1 × 10^6^ cells per well. A single colony of GBS strain HB31 was picked and cultured in 5 mL of broth at 37 °C for 24 h. Two milliliters of the bacterial suspension was then centrifuged at 6000 rpm for 5 min, resuspended in PBS, and diluted using a McFarland turbidity standard to a concentration of 1 × 10^7^ CFU/mL. Baicalein solutions were prepared at concentrations of 512, 256, 128, 64, 32, 16, 8, 4, and 2 μg/mL and were incubated with the HB31 suspension (1 × 10^7^ CFU/mL) for 4 h. The positive control group consisted of HB31 alone, and the blank control group consisted of PBS alone. After removing the complete medium from the 24-well plates, the wells were washed three times with PBS. Meanwhile, the incubated bacterial solutions were centrifuged at 3000 rpm for 5 min, resuspended in an equal volume of antibiotic-free DMEM high glucose medium, and then added to the washed 24-well plates. Six replicate wells were prepared for each condition, and the plates were placed in a 37 °C incubator for 2 h to allow infection. The multiplicity of infection (MOI) at this stage was 10. Following the infection period, the culture medium was discarded, and the wells were washed once with PBS. A PBS solution containing 100 μg/mL gentamicin (Aladdin, Shanghai Aladdin Biochemical Technology Co., Ltd., Shanghai, China) was then added, and the plates were incubated at 37 °C for an additional 2 h to kill extracellular bacteria. Subsequently, 200 μL of 1% Triton X-100 (Beijing Solarbio Science & Technology Co., Ltd., Beijing, China) was added to each well to lyse the cells for 5 min. The lysates were serially diluted and plated onto LB agar plates supplemented with 5% FBS. Colony-forming units (CFU) were counted after overnight incubation, and bacterial invasion efficiency was determined using the plate counting method.

For the invasion assay conducted without prior incubation with baicalein, baicalein was prepared at concentrations of 512, 256, 128, 64, 32, and 16 μg/mL and incubated with HB31 (1 × 10^7^ CFU/mL) for 0 h; the positive control group consisted of HB31 alone, and the blank control group consisted of PBS. For the invasion assay in which baicalein was first incubated with bMECs for 1 h, baicalein at the same concentrations was incubated with bMECs for 1 h, followed by the addition of HB31 at 1 × 10^7^ CFU/mL. Subsequent steps for both assays were consistent with the invasion assay described above. The invasion rate was calculated as follows: Invasion rate = (Number of intracellular GBS/Total number of GBS added) × 100%.

#### 2.3.2. Assay for the Inhibition of GBS Adhesion by Baicalein

bMECs were treated with GBS HB31 in an invasion assay. Baicalein was prepared at concentrations of 512, 256, 128, 64, 32, and 16 μg/mL and incubated with HB31 (1 × 10^7^ CFU/mL) for 4 h. The positive control group consisted of HB31 alone, and the blank control group consisted of PBS. After removing the complete medium from the 24-well plates, the wells were washed three times with PBS. Meanwhile, the incubated bacterial solution was centrifuged at 3000 rpm for 5 min, resuspended in an equal volume of antibiotic-free DMEM high glucose medium, and added to the washed 24-well plates. Six replicates were prepared, and the plates were placed in a 37 °C incubator for 2 h to allow infection, with a MOI of 10. Subsequently, the culture medium was discarded, and the wells were washed six times with PBS. Then, 200 μL of 1% Triton X-100 was added to each well to lyse the cells for 5 min. The lysate was serially diluted, plated onto LB agar plates containing 5% serum, and CFUwere counted after overnight incubation. The adhesion rate was calculated as follows: Adhesion rate = (Number of GBS cells adhering to host cells/Total number of GBS cells added) × 100%.

### 2.4. Assessment of the Effect of Baicalein on the Survival Rate of bMECs

The Cell Counting Kit-8(CCK-8) assay (Beijing Solarbio Science & Technology Co., Ltd., Beijing, China) was used to assess cell viability. bMECs in the logarithmic growth phase were harvested by trypsinization and counted. The cell density was adjusted, and the cell suspension was seeded into 96-well plates at 100 μL per well. The seeding density was predetermined based on preliminary experiments: typically 1000–5000 cells/well for proliferation assays and 5000–10,000 cells/well for cytotoxicity assays. Each group contained three replicate wells. Blank control wells (containing medium only, without cells) and negative control wells (containing cells without drug treatment) were also included. The plates were incubated overnight at 37 °C in a 5% CO_2_ incubator to allow cell adherence. Subsequently, the culture medium was discarded, and 100 μL of fresh medium containing various concentrations of baicalein (2, 4, 8, 16, 32, 64, 128, 256, 512, and 1024 μg/mL) was added to the wells. bMECs were incubated with these different concentrations of baicalein for 2 h and 24 h, respectively. After incubation, 10 μL of CCK-8 solution was added to each well (avoiding bubble formation), and the plates were gently agitated to ensure mixing. Following an additional 1 h incubation (the duration was predetermined to ensure that the OD value of the control group reached 0.8–1.2), the absorbance (OD value) of each well was measured at 450 nm using a microplate reader, with a reference wavelength set at 600–650 nm. Cell viability was calculated using the following formula: Cell viability (%) = (OD experimental group − OD blank group)/(OD negative control group − OD blank group) × 100%. The experiment was repeated three times, and the results were expressed as mean ± standard deviation.

### 2.5. Metabolomic Sequencing of GBS

#### 2.5.1. Metabolites Extraction

After overnight incubation of GBS HB31, the bacterial pellet was obtained by centrifugation, washed with PBS, and resuspended in PBS. The suspension was then divided into two groups: the baicalein group (co-incubated with 16 μg/mL baicalein for 4 h) and the control group (PBS suspension of HB31). Following incubation, the bacterial pellet was collected by centrifugation, immediately frozen in liquid nitrogen for 30 s, and stored at −80 °C as metabolomics samples. The LC/MS system used for metabolomics analysis consisted of a Waters Acquity I-Class PLUS (Waters Corporation, Milford, MA, USA) ultra-high performance liquid chromatography system coupled with a Waters Xevo G2-XS QTof high-resolution mass spectrometer (Waters Corporation, Milford, MA, USA). Chromatographic separation was performed on a Waters Acquity UPLC HSS T3 (Waters Corporation, Milford, MA, USA) column (1.8 μm, 2.1 × 100 mm). The mobile phases were as follows: in positive ion mode, mobile phase A was 0.1% formic acid in water and mobile phase B was 0.1% formic acid in acetonitrile; the same mobile phase composition was used in negative ion mode. The injection volume was 2 μL.

#### 2.5.2. LC-MS/MS Analysis

Metabolomics data analysis was conducted by BestMS Technologies Co., Ltd. (Qingdao, China). The Waters Xevo G2-XS QTOF high-resolution mass spectrometer was used to collect primary and secondary mass spectrometry data in MSe mode, controlled by the acquisition software (MassLynx V4.2, Waters Corporation, Milford, MA, USA)). In each data acquisition cycle, dual-channel data acquisition was performed simultaneously at low collision energy (off) and high collision energy (ranging from 10 to 40 V). The scan frequency was set to 0.2 s per mass spectrum. The parameters of the ESI ion source were as follows: capillary voltage: 2500 V (positive ion mode) or −2000 V (negative ion mode); cone voltage: 30 V; ion source temperature: 100 °C; desolvation gas temperature: 500 °C; cone gas flow rate: 50 L/h; desolvation gas flow rate: 800 L/h.

#### 2.5.3. Metabolomics Data Analysis

The raw data collected using MassLynx V4.2 were processed with Progenesis QI software V3.0 (Waters Corporation, Milford, MA, USA)for peak extraction, peak alignment, and other data processing operations. Compound identification was performed based on the online METLIN database and a self-built library within the Progenesis QI software. After normalizing the original peak area information to the total peak area, subsequent analyses were carried out. Principal component analysis (PCA) and Spearman correlation analysis were used to assess the repeatability of within-group samples and quality control samples. The identified compounds were searched for classification and pathway information in the KEGG (Kyoto Encyclopedia of Genes and Genomes), HMDB (Human Metabolome Database), and LipidMaps databases. Based on the grouping information, fold changes were calculated and compared, and a *t*-test was used to determine the significance *p*-value for each compound. The R package “ropls” was used to perform orthogonal partial least squares discriminant analysis (OPLS-DA), and 200 permutation tests were conducted to verify the reliability of the model. The variable importance in projection (VIP) value of the model was calculated using multiple cross-validation. Differential metabolites were screened using a combination of fold change, *p*-value, and VIP value from the OPLS-DA model. The screening criteria were FC > 1, *p* < 0.05, and VIP > 1. The significance of KEGG pathway enrichment for differential metabolites was calculated using a hypergeometric distribution test.

### 2.6. Transcriptome Sequencing of GBS

#### 2.6.1. RNA Extraction and Library Preparation and Sequencing

After overnight incubation of GBS HB31, the bacterial pellet was obtained by centrifugation, washed with PBS, resuspended in PBS, and divided into two groups: the baicalein group (co-incubated with 16 μg/mL baicalein for 4 h) and the control group (PBS suspension of HB31). The pellet was collected after centrifugation, frozen in liquid nitrogen for 30 s, and then stored at −80 °C as transcriptomic samples.

Total RNA was extracted from the samples using the Kangwei Tissue RNA Extraction Kit (Beijing Kangwei Century Biotechnology Co., Ltd., Beijing, China). High-quality RNA samples were used for subsequent library preparation. The RiboCop rRNA Depletion Kit for Mixed Bacterial Samples (Lexogen, Inc., Greenland, NH, USA) was employed to remove ribosomal RNA. The mRNA was randomly fragmented into small fragments of approximately 200 bp. Using the mRNA as a template, double-stranded cDNA was synthesized via reverse transcription with random primers. During second-strand cDNA synthesis, dUTP was substituted for dTTP. The resulting double-stranded cDNA was treated with End Repair Mix to create blunt ends, followed by 5′-end phosphorylation and 3′-end A-base addition. Y-shaped sequencing adapters were then ligated. Subsequently, UNG enzyme was used to eliminate the dUTP-containing second strand of cDNA, ensuring that the library contained only the first strand of cDNA. RNA library preparation was performed using the Illumina^®^ Stranded mRNA Prep Ligation kit (Illumina, San Diego, CA, USA). RNA-seq paired-end sequencing was conducted on an Illumina NovaSeq 6000 (Illumina, Inc., San Diego, CA, USA).

#### 2.6.2. Transcriptome Sequencing Data Analysis

Raw reads were further processed using the bioinformatics analysis platform BMKCloud (www.biocloud.net). Raw data in Fastq format were initially processed via an internal Perl script. This step involved quality control by removing sequences containing adapters, poly-N sequences, and low-quality reads from the raw data. Subsequently, these sequences were aligned against BGI’s ribosomal databases (constructed from NCBI and Silva sources) using SOAP to remove ribosomal sequences. Concurrently, Q20, Q30, and GC content metrics were calculated for the remaining valid data. All downstream analyses were based on this high-quality cleaned data. Bowtie2 was employed to align the valid data to the reference genome sequence. RSEM was used for gene expression quantification, yielding count, TPM, and FPKM values. DESeq2 was employed for differential expression analysis between the two groups. DESeq2 uses a negative binomial distribution-based model to identify differentially expressed genes within the expression data. The Benjamini–Hochberg method was applied to control the false discovery rate (FDR), yielding adjusted *p*-values. Genes with adjusted *p*-values < 0.01 and a fold change ≥ 2 from the DESeq2 analysis were designated as differentially expressed (actual thresholds may vary; refer to the report for specifics). Differential expression analysis was also performed on the two samples using edgeR, with thresholds of FDR < 0.01 and fold change ≥ 2 set for significance (actual thresholds may be adjusted; refer to the report for details).KEGG is a database resource for understanding the higher-level functions and utilities of biological systems (such as cells, organisms, and ecosystems) from molecular-level information, particularly large-scale molecular datasets generated through genome sequencing and other high-throughput experimental techniques (http://www.genome.jp/kegg/ (accessed on 13 December 2025)). We employed the KOBAS database and cluster Profiler software to analyze the enrichment of differentially expressed genes within KEGG pathways [28]. Structural analysis was conducted using Rockhopper software to obtain information on transcription start and termination sites, operons, and UTRs.

### 2.7. Detection of Resistance and Virulence Genes

Total RNA was extracted from the baicalein group and control group using TRIzol reagent (Invitrogen, Thermo Fisher Scientific, Carlsbad, CA, USA). For the baicalein group, baicalein was diluted to a concentration of 8 μg/mL in PBS. GBS HB31 with an OD_600_ > 0.4 was centrifuged, and the pellet was incubated with the baicalein solution for 4 h. For the control group, the HB31 pellet was incubated with PBS for 4 h.

Total RNA was then reverse transcribed using the PrimeScript™ RT Reagent Kit with gDNA Eraser Kit (Takara Biomedical Technology, Beijing, China), and the experiments were performed according to the manufacturer’s instructions. Primers were designed using Primer 5.0 software based on gene sequences obtained from the GenBank database. The primer sequences were submitted to BGI Shenzhen for synthesis and are shown in Table 1. mRNA expression was measured using the SYBR Green mix kit (Takara Biomedical Technology, Beijing, China) on an ABI 7500 Fast instrument according to the manufacturer’s instructions, with *16sRNA* as a reference gene. Relative quantification of gene amplification by Quantitative real-time PCR was performed using cycle threshold (Ct) values. The relative mRNA expression of the selected genes was normalized to the control gene *16S rRNA* and determined using the 2^−ΔΔCt^ method.

### 2.8. Assay for Virulence and Antibiotic Resistance Gene Expression in bMECs

Total RNA was extracted using Trizol reagent. bMECs were seeded into 6-well plates at a density of 10^6^ cells/well and cultured overnight, then washed twice with PBS before use. HB31 strain was cultured overnight, centrifuged, diluted with PBS to 10^7^ CFU/mL, and incubated with or without baicalein (8 μg/mL) for 4 h. After centrifugation, the bacterial pellets were resuspended in DMEM high-glucose medium. The experiment consisted of four groups: control group (DMEM high-glucose medium only), HB31 group, HB31+baicalein group, and baicalein group. The prepared media of each group were added to the washed bMECs for incubation. Incubation time was 4 h for antioxidant index measurement and 12 h for inflammatory factor measurement (both determined as optimal in preliminary experiments). Subsequently, total RNA was reverse transcribed using the PrimeScript™ RT Reagent Kit with gDNA Eraser Kit (Takara Biomedical Technology, Beijing, China), and all operations were performed according to the manufacturer’s instructions. Primers were designed using Primer 5.0 software based on gene sequences retrieved from the GenBank database, and the sequences were synthesized by BGI Shenzhen. The primer sequences are shown in Table 2. mRNA expression was detected using the SYBR Green mix kit (Takara Biomedical Technology, Beijing, China) on an ABI 7500 Fast instrument according to the manufacturer’s instructions, with *β-actin* serving as the reference gene. Relative quantification of gene amplification by Quantitative real-time PCR was performed using cycle threshold (Ct) values. The relative mRNA expression levels of the selected genes were normalized to the control gene *β-actin* and calculated using the 2^−ΔΔCt^ method.

### 2.9. Data Processing

The data were analyzed using SPSS 26 (IBM-SPSS Inc., Chicago, IL, USA). Measurement data are presented as mean ± standard deviation (SD). Comparisons between two groups were analyzed using a two-tailed *t*-test, and a *p*-value < 0.05 was considered statistically significant. The figures were generated using Prism 8.0 (GraphPad Software Inc., San Diego, CA, USA) and ChiPlot (https://www.chiplot.online/ (accessed on 12 January 2026)).

## 3. Result

### 3.1. Baicalein Inhibits the Invasion and Adhesion of GBS

**Figure 1:**
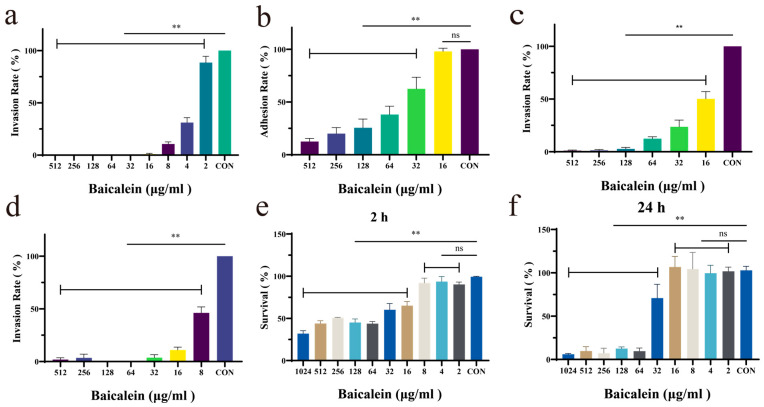
(**a**) Invasion results of GBS HB31 after 4 h of incubation with baicalein. (**b**) Adhesion results of GBS HB31 after 4 h of incubation with baicalein. (**c**) Invasion results of GBS HB31 without pre-incubation with baicalein. (**d**) Invasion results of HB31 after 1 h of incubation of bMECs with baicalein. (**e**) Survival rate of bMECs after 2 h of treatment with baicalein. (**f**) Survival rate of bMECs after 24 h of treatment with baicalein. ** *p* < 0.01 vs. control group. ns = not significant.

Based on preliminary experiments, an invasion time of 2 h was used in this study, and a 4 h pre-incubation of baicalein with GBS strain HB31 was found to provide optimal inhibition. At concentrations from 4 to 512 μg/mL, baicalein significantly inhibited GBS HB31 invasion, with invasion rates consistently below 50% (Figure 1a). Adhesion assays showed that baicalein also effectively suppressed GBS adhesion in a concentration-dependent manner (Figure 1b). To assess the potential cytotoxicity of baicalein to bMECs during the 2 h invasion and adhesion assays, we found that baicalein concentrations below 512 μg/mL resulted in a bMEC survival rate of over 50% after 2 h, indicating that its anti-invasion effect was not due to cytotoxicity (Figure 1e). Under 24 h exposure, bMECs maintained high viability at baicalein concentrations below 32 μg/mL; notably, baicalein at 4 μg/mL already significantly inhibited GBS invasion (Figure 1d). Based on these observations, we hypothesized that baicalein might act directly on GBS even without pre-incubation (Figure 1c). To test this, GBS was co-incubated with bMECs in the presence of various baicalein concentrations. The results confirmed that baicalein retained its inhibitory activity against GBS invasion under these conditions. Furthermore, pre-incubating bMECs with baicalein before adding GBS produced a stronger inhibitory effect than adding baicalein and GBS simultaneously. Together, these findings suggest that baicalein may act on both GBS and bMECs, possibly by activating specific signaling pathways in bMECs, supporting its potential clinical application.

### 3.2. MIC and MBC of Baicalein Against HB31

**Figure 2:**
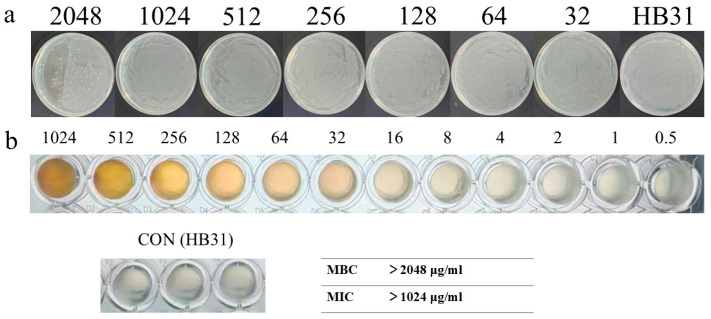
(**a**) MBC results for baicalein against HB31. (**b**) MIC results for baicalein against HB31.

MIC of baicalein against GBS HB31 was determined. Baicalein at 2048 μg/mL significantly inhibited the growth of GBS HB31, although a subpopulation of HB31 cells survived (Figure 2a). Growth inhibition was observed between 12 and 20 h of measurement. At 24 h, growth was restored in the presence of 512 and 1024 μg/mL baicalein, whereas inhibition persisted at 2048 μg/mL. These results suggest that HB31 may adapt to the selective pressure exerted by moderate concentrations of baicalein, while excessively high concentrations remain insurmountable. MBC of baicalein against HB31 was >1024 μg/mL (Figure 2b).

### 3.3. Differential Analysis of Metabolites Produced by GBS Treated with Baicalein

**Figure 3:**
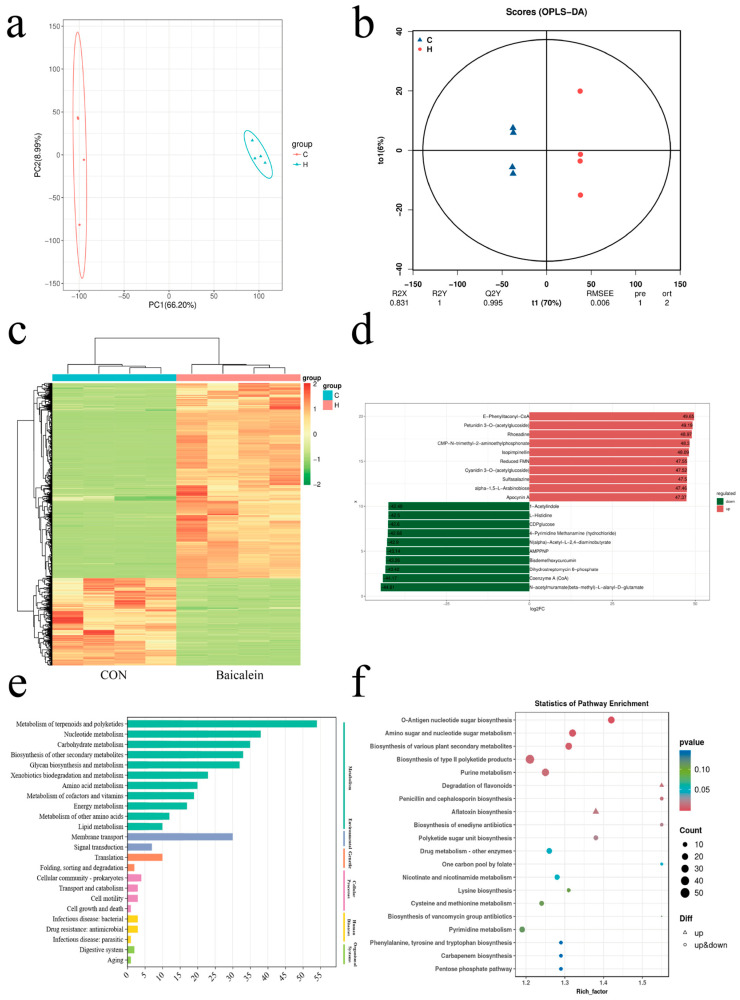
(**a**) Principal component analysis (PCA) plot comparing the baicalein group and the HB31 group (control). (**b**) Orthogonal partial least squares discriminant analysis (OPLS-DA) plot used to assess model stability. (**c**) Heatmap of metabolite differences between the baicalein group and the HB31 group (control). (**d**) log_2_FC results for the top 10 upregulated and downregulated metabolites in the experimental group compared to the control group (HB31 group) after log transformation of the fold changes in differentially expressed metabolites. (**e**) Differential KEGG (Kyoto Encyclopedia of Genes and Genomes) Level 2 classification plot between the baicalein group and the HB31 group (control group). (**f**) Bubble plot of differences in KEGG-annotated pathways between the baicalein group and the HB31 group (control group). Each point in the figure represents a KEGG pathway, and the size of the circle indicates the number of differentially expressed metabolites enriched in that pathway; the larger the circle, the more differentially expressed metabolites are present.

To further investigate the effects of baicalein on GBS at the intracellular metabolic level, a non-targeted metabolomics analysis was performed. Principal component analysis (PCA) revealed complete separation between the control group and the baicalein-treated group, indicating good independence (Figure 3a). Generally, a Q^2^Y value greater than 0.5 indicates a valid model, while a Q^2^Y value above 0.9 suggests an excellent model. The observation that the blue points were generally located above the red points indicated that the training and testing sets were well balanced. In this study, the Q^2^Y value was 0.995. Differentially expressed metabolites can be screened based on variable importance in projection (VIP) analysis, further confirming the reliability of the model. Collectively, these results demonstrate significant differences between the control group and the baicalein-treated group (Figure 3b). The clustering heatmap revealed that the metabolic changes in the baicalein-treated group were highly significant. A total of 833 differential metabolites were identified in the baicalein-treated group, of which 565 were upregulated and 268 were downregulated (Figure 3c). Most of the upregulated metabolites were generated from the metabolism of baicalein itself. The significantly downregulated metabolites included Acetylindole, L-Histidine, CDPglucose, Pyrimidine Methanamine (hydrochloride), N(alpha)-Acetyl-L-2,4-diaminobutyrate, AMPPNP, Bisdemethoxycurcumin, Dihydrostreptomycin 6-phosphate, Coenzyme A (CoA), and N-acetylmuramate (beta-methyl)-L-alanyl-D-glutamate (Figure 3d).

Based on the metabolomics results, we performed enrichment analysis on the secondary classifications of metabolites from GBS HB31 following baicalein treatment. The analysis revealed effects on Metabolism, Genetic Information Processing, Environmental Information Processing, Cellular Processes, Organismal Systems, and Human Diseases. The most pronounced effects were observed in Metabolism, Nucleotide Metabolism, Carbohydrate Metabolism, Glycan Biosynthesis, Amino Acid Metabolism, and Membrane Transport (Figure 3e,f). Baicalein treatment downregulated the metabolism of lysine (Lys), histidine (His), proline (Pro), tyrosine (Tyr), glutamate (Glu), aspartate (Asp), alanine (Ala), valine (Val), and tryptophan (Trp) in HB31. Nucleic acid metabolism-related metabolites, including adenine, uridine monophosphate (UMP), and 2′-deoxyadenosine 5′-monophosphate (dAMP), as well as phosphoenolpyruvate (PEP), were also downregulated. Additionally, the increased NADH/NAD^+^ ratio and decreased ATP levels indicated that baicalein inhibited the energy metabolism of GBS HB31. Collectively, these results suggest that baicalein affects the metabolism of GBS HB31, thereby inhibiting DNA replication, transcription, gene expression, and carbohydrate metabolism.

### 3.4. Differential Analysis of Transcriptome Produced by GBS Treated with Baicalein

**Figure 4:**
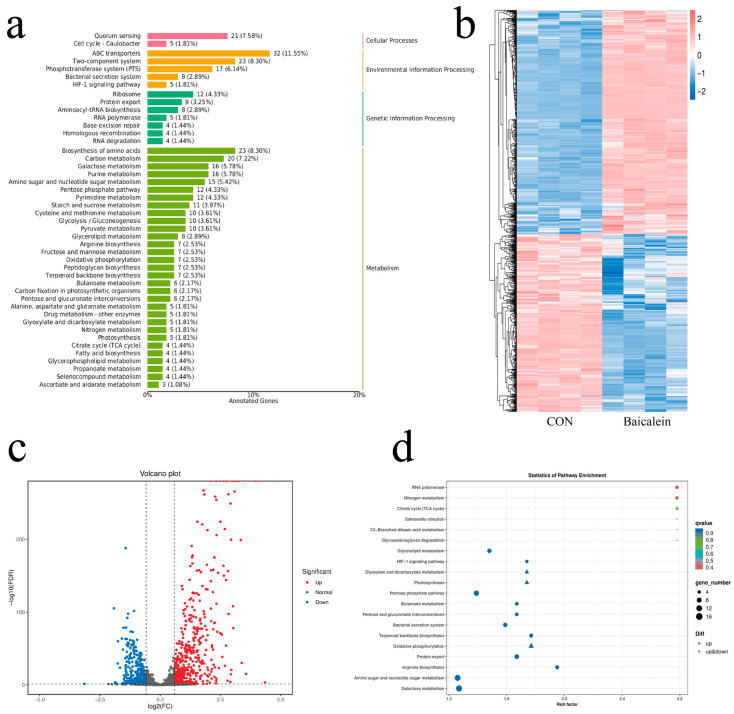
(**a**) Differential KEGG Level 2 classification map between the baicalein group and the HB31 group (control). (**b**) Heatmap between the baicalein group and the HB31 group (control). (**c**) Volcano plot of differential expression between the baicalein group and the HB31 group (control) (**d**) Differential KEGG-annotated pathway bubble plot of transcriptional differences between the baicalein group and the HB31 group (control).

To further investigate the mechanism by which baicalein inhibits GBS invasion of bMECs, a transcriptomic analysis was performed. The results showed that baicalein had the most pronounced effects on GBS quorum sensing, ABC transporters, two-component systems, phosphotransferase systems, amino acid biosynthesis, and carbon metabolism. It also modulated pathways including the pentose phosphate pathway, glycerolipid metabolism, amino sugar and nucleotide sugar metabolism, and galactose metabolism, while inhibiting GBS glyoxylate and dicarboxylate metabolism as well as oxidative phosphorylation (Figure 4a). Baicalein significantly affected the gene regulation of HB31. Following treatment, a total of 820 genes were differentially expressed in HB31, of which 362 were downregulated and 458 were upregulated (with |log_2_(FC)| > 1 and *p* < 0.05 as the thresholds for identifying differentially expressed genes; Figure 4b,c). The differential metabolites in the baicalein-treated group were significantly enriched in multiple pathways, including nitrogen metabolism, the TCA cycle, oxidative phosphorylation, RNA polymerase, the HIF-1 signaling pathway, the pentose phosphate pathway, amino sugar and nucleotide sugar metabolism, and galactose metabolism (Figure 4d).Collectively, these results suggest that baicalein may inhibit GBS invasion and adhesion to host cells by suppressing GBS energy metabolism and quorum sensing systems, while interfering with its carbon, nitrogen, amino acid, and sugar metabolism.

### 3.5. Differential Gene and Metabolite Expression in GBS After Baicalein Treatment

Integrating the transcriptomic and metabolomic results, baicalein significantly affected the virulence and physiological state of GBS. At the level of virulence-related genes, baicalein upregulated the expression of *cylE*, *scpB*, and the two-component system genes *vicK*, *vicR*, and *saeS*, while downregulating the adhesion-related gene *pbsP* as well as *hylB* and *cfb*. In addition, *lrgAB* expression was significantly reduced, although its upstream regulator LytST showed no significant change [29]. At the metabolic level, baicalein induced downregulation of ATP, NAD^+^ and PEP, a marked upregulation of NADH, and an increase in acetoin, a downstream product of pyruvate metabolism (Table 3). These results suggest that baicalein may impair the invasion and adhesion ability of GBS to host cells by disrupting bacterial energy metabolism (characterized by decreased ATP, an imbalanced NADH/NAD^+^ ratio, and reduced PEP), altering the metabolic flux of pyruvate (promoting acetoin production), and synergistically regulating key virulence genes (e.g., suppressing the expression of *pbsP*, *hylB*, and *cfb*).

### 3.6. Validation of Transcriptome-Associated Virulence Genes

**Figure 5:**
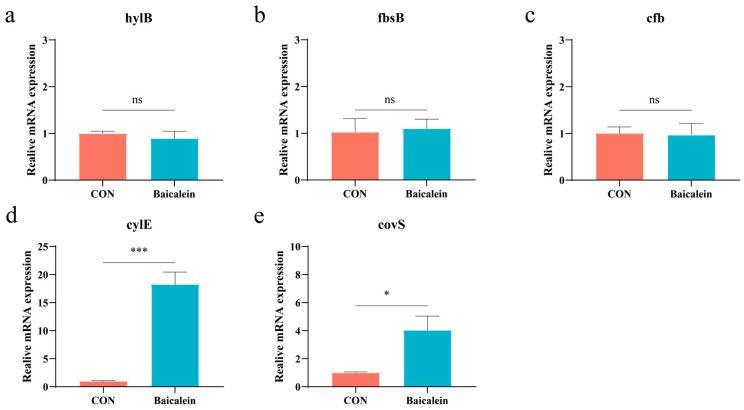
(**a**–**e**) The mRNA expression levels of hylB, fbsB, cfb, cylE, and covS are shown. * *p* < 0.05 or *** *p* < 0.001 vs. control group. ns = not significant.

**Figure 6:**
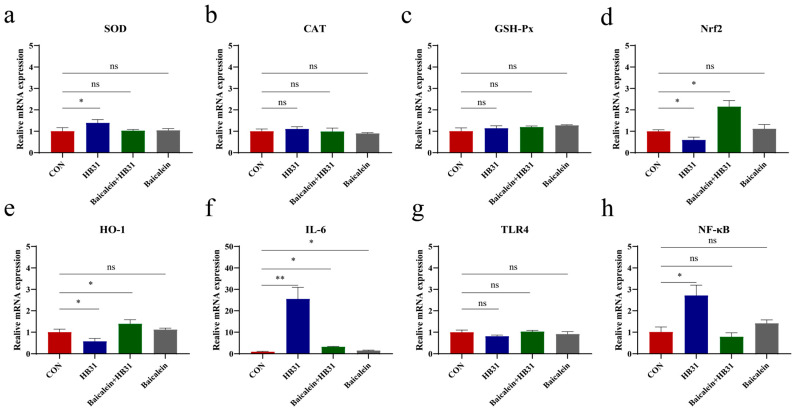
(**a**–**e**) Expression levels of antioxidant-related genes (*SOD*, *CAT*, *GSH-Px*, *Nrf2*, *HO-1*) and inflammatory-related genes (*IL-6*, *TLR4*, *NF-κB*) in bMECs were examined. The results are shown in panels (**f**–**h**). * *p* < 0.05 or ** *p* < 0.01 vs. control group. ns = not significant.

Based on the transcriptomic results, we found that baicalein significantly influenced the regulation of virulence genes in HB31. Therefore, we re-validated and measured common virulence genes. Under baicalein treatment, there were no significant changes in *hylB, fbsB*, and *cfb*, although the mRNA expression of *hylB* and *cfb* showed a decreasing trend without statistical significance (Figure 5a–c). Baicalein significantly increased the mRNA expression of *cylE* and *covS* (*p* < 0.05) (Figure 6d,e). The significant upregulation of *cylE* is attributed to the antioxidant role of hemolysin in GBS [30]. Additionally, *tpx* and *recJ* in GBS, which encode a putative thioperoxidase and a single-strand DNA exonuclease, respectively, were both upregulated according to the transcriptomic data, thereby promoting DNA repair under oxidative stress [31,32]. It is speculated that baicalein induced oxidative stress in HB31, leading to the upregulation of *cylE* and the dTDP-L-rhamnose metabolic pathway. This facilitates the synthesis of L-rhamnose, which is an important precursor for β-hemolysin synthesis [33].

### 3.7. Baicalein Inhibits Oxidative Stress and Inflammation Induced by HB31

Given that baicalein effectively reduces the invasive activity of GBS HB31 and possesses anti-inflammatory and antioxidant properties, we measured the mRNA expression of antioxidant and inflammation-related genes in bMECs following GBS infection. The results showed that baicalein reduced the mRNA expression of *SOD* induced by GBS HB31 (Figure 6a). HB31 increased the mRNA expression of *GSH-Px* and *CAT*, but the differences were not significant (Figure 6b,c). GBS HB31 significantly downregulated the mRNA expression of *Nrf2* and *HO-1*, whereas baicalein significantly upregulated their expression (Figure 6d,e). The markedly increased *IL-6* mRNA expression induced by HB31 indicated an inflammatory response in bMECs, and the addition of baicalein effectively reduced this *IL-6* mRNA expression (Figure 6f). HB31 showed a tendency to decrease *TLR4* mRNA expression, but the difference was not significant. Meanwhile, HB31 significantly increased the mRNA expression of *NF-κB*, and the addition of baicalein significantly reduced its expression (Figure 6g,h). The significant increases in *IL-6* and *NF-κB* indicated that HB31 induced an inflammatory response in bMECs, and baicalein was able to effectively alleviate this inflammatory response.

## 4. Discussion

GBS causes invasive bovine mastitis [34]. The pathogen’s ability to invade the host relies primarily on the regulation of virulence factors that enable adhesion, invasion, and immune evasion, thereby ensuring successful invasion and establishment within the host [35]. During GBS invasion, bMECs are the first cells in the mammary gland to be attacked, and their high metabolic rate during lactation contributes to the persistence of mastitis [36,37]. Therefore, in this study, bMECs were selected as the test cells for GBS invasion and adhesion assays derived from dairy cows to investigate the mechanism by which baicalein inhibits GBS invasion.

Our experimental results indicate that baicalein inhibits the invasion of bMECs under three different conditions: without pre-incubation with HB31, after 1 h of pre-incubation with bMECs, and after 4 h of pre-incubation with HB31. All three conditions showed concentration-dependent inhibition with similar effects, suggesting that baicalein likely acts on both HB31 and bMECs simultaneously. Studies have shown that baicalein can protect mice from lung damage caused by Streptococcus pneumoniae [14]. Additionally, we measured the survival rate of bMECs in the presence of baicalein. Within 2 h, baicalein at concentrations that effectively inhibit the invasion of Streptococcus agalactiae exhibited no cytotoxic effects on bMECs. After 24 h of treatment, the concentration threshold at which baicalein exhibits cytotoxicity to bMECs was above 64 μg/mL, which may be related to the inherent characteristics of bMECs as primary cells. Nevertheless, previous studies have reported findings similar to ours: baicalein at concentrations below 0.25 mM (approximately 67.6 μg/mL) had no adverse effects on the growth of human corneal epithelial cells (HCECs) [38], Furthermore, another study showed that baicalein, within the concentration range of 10 to 100 μM (approximately 2.7 to 27 μg/mL), led to an increase in cell viability over time during a 96 h treatment period in human periodontal ligament cells (PDLCs). Therefore, it is speculated that extending the incubation time of baicalein with bMECs may further enhance the viability of bMECs [39].

Given that baicalein can reduce the invasion and adhesion of GBS while possessing antioxidant and anti-inflammatory properties, we sought to investigate its effects on host cells during bacterial invasion. To this end, this study measured antioxidant and inflammatory markers in bMECs following GBS invasion. *Nrf2* serves as the primary cellular sensor for oxidative stress and activates the downstream gene *HO-1* [40,41]. Activation of the Nrf2-HO-1 signaling pathway counteracts inflammation. Specifically, activation of the Nrf2 signaling pathway can inhibit NF-κB activity and reduce inflammatory responses [36,42], and has been reported to reduce the production of cytokines such as TNF, IL-1β, IL-6, and IL-8 [43]. The results of this study indicate that GBS HB31 suppresses the mRNA expression of *Nrf2* and *HO-1*. The addition of baicalein significantly increased the mRNA expression of both genes, suggesting activation of the Nrf2 pathway. Furthermore, baicalein significantly reduced the mRNA expression of *NF-κB* and *IL-6*, indicating that baicalein can effectively alleviate the inflammatory response induced by GBS. Under the influence of baicalein, both the MIC and MBC of GBS HB31 were >1024 μg/mL, indicating that baicalein alone does not exert a bacteriostatic effect on GBS. This raises the question of why it led to a reduction in the invasiveness of GBS HB31. We hypothesize that this is due to baicalein reducing the expression of GBS virulence factors. To test this hypothesis, we conducted metabolomic and transcriptomic analyses of GBS treated with baicalein.

Various stimuli encountered by GBS in its growth environment—including carbon sources, the availability of external heme, and pressure from other drugs—can influence the metabolic pathways governing its growth and survival [44]. Studies have shown that GBS treated with linalool may utilize large amounts of amino acids as a carbon source to adapt to harsh living conditions [45]. Furthermore, amino acids such as alanine, as key components of cellular structure, can be used to repair damaged cellular structures [8]. Under the influence of baicalein, the metabolism of amino acids such as proline, alanine, and aspartic acid is downregulated. It is speculated that GBS maintains its metabolism by reducing or utilizing these amino acids as carbon sources to resist the stress induced by baicalein. The most significantly affected metabolic pathway is nucleic acid metabolism. The normal functioning of nucleic acid metabolism and gene transcription is fundamental to the survival and metabolism of GBS [46]. Under the influence of baicalein, the levels of adenine and uridine monophosphate (UMP) in HB31 are reduced, indicating that baicalein inhibits DNA replication, transcription, and gene expression in HB31. Increasing the dosage or duration of baicalein treatment would likely cause nucleotide metabolism disorders, leading to cellular damage or even cell death [47]. In addition to amino acid and nucleotide metabolism, energy acquisition is also critical for GBS. Since GBS lacks the enzymes required for the tricarboxylic acid (TCA) cycle [48], the pyruvate produced is metabolized through the following three pathways: (a) Under anaerobic conditions, the primary fermentation product is lactic acid, but it also contains significant amounts of ethanol, acetate, acetion, and diacetyl [49]. Under aerobic conditions, the production of acetion, diacetyl, and acetate exceeds that of ethanol. (b) In the absence of external heme or menadione (under non-permissive respiratory conditions), lactate remains the primary fermentation product, leading to a significant drop in pH. (c) When external heme and menadione are added in the presence of oxygen (under conditions where respiration is permitted), the respiratory chain (NDH-2/cyt bd) regains function. Metabolism shifts toward the production of acetate, acetion, and diacetyl, reducing the amount of pyruvate available for conversion to lactate [44,50]. In our experiments, where heme and menadione were not added, HB31 was unable to rapidly generate large amounts of energy [51,52]. Under these conditions, HB31 could only produce energy through fermentation; growth required not only ATP production but also a mechanism to recycle NADH back to NAD^+^ to allow glycolysis to continue [50]. Our results indicate that, due to the presence of baicalein, the upstream metabolite of pyruvate, PEP, is downregulated. This appears to signal that HB31’s overall pyruvate metabolism is inhibited. The observed increase in the NADH/NAD^+^ ratio is correlated with the decrease in ATP levels. This may reflect an inhibition of the oxidation of NADH to NAD^+^ in GBS. However, whether this association directly causes the energy metabolism disturbance or represents a concomitant phenomenon under stress conditions remains to be further explored through functional experiments, such as directly measuring the activity of NAD^+^/NADH oxidoreductases.

Invasion is a critical capability of GBS and is often used as an indicator of its virulence. GBS possesses numerous virulence factors that influence invasion, such as fibrinogen-binding protein (Fbs), high-virulence GBS adhesin (HvgA), and laminin-binding protein (Lmb). These virulence factors are generally regulated by two-component systems, such as CovRS and SaeRS. The VicRK signaling system plays a conserved role as a key regulator of bacterial oxidative stress responses [53], acidification, cell wall metabolism, and biofilm formation [54]. The VicRK system has received extensive attention in Streptococcus mutans research but has been studied relatively less in GBS. However, based on studies of Streptococcus mutans, the VicRK system influences virulence by regulating bacterial physiological states [54]. The upregulation of *vicK* observed in our transcriptomic results is hypothesized to be an attempt by GBS to maintain its own physiological state under baicalein stress, thereby restoring virulence. Among our transcriptomic results, the downregulation of *pbsP* was the most significant change, and the concurrent significant upregulation of its regulatory gene *saeR* warrants particular attention. The SaeRS system is a dynamic and highly specialized regulatory system capable of modulating the PbsP adhesin, facilitating penetration of the host barrier, and enabling these bacteria to survive within the host during lethal infections [55]. PbsP plays a role in promoting systemic transmission and invasive infection by GBS [56]. In a mouse model, when GBS needs to maintain vaginal colonization, *pbsP* is highly upregulated under the control of SaeRS, and *pbsP* transcription in the vagina is significantly increased [57]. CovRS suppresses expression at the *pbsP* locus [58], but direct binding of CovR to the *pbsP* promoter has not been demonstrated, suggesting that this regulation may be indirect [56]. In a mouse vaginal colonization model, the SaeRS system is strongly activated in vivo, leading to overexpression of the virulence factors PbsP and BvaP [55]. Transcriptomic results indicate that, under the action of baicalein, *pbsP* gene expression is significantly reduced, while *saeR* and *saeS* gene expression increases; this may be the result of indirect regulation by CovRS. Knocking out *covRS* leads to a significant increase in adhesion to host epithelial cells [59,60], which is consistent with the increased expression of *covS* mRNA observed in our quantitative RT PCR results. Based on the transcriptomic data, we hypothesize that baicalein may indirectly downregulate *pbsP* through CovRS, thereby inhibiting the adhesion and invasion capabilities of GBS. This hypothesis awaits further validation through *pbsP* gene knockout/complementation experiments and animal infection models. Targeting the *pbsP* pathway may represent a potential direction for anti-virulence strategies.

At non-inhibitory concentrations, natural compounds can inhibit GBS by disrupting its metabolism and reducing its virulence. For example, baicalein inhibits hemolysin synthesis by interfering with energy and carbohydrate metabolism [33], while matrine effectively reduces the virulence of GBS by inhibiting bacterial adhesion, lowering whole-blood survival rates, and suppressing biofilm formation and persistence [61]. The metabolic pathways selected by pathogens are not always the most energy-efficient; rather, they prioritize pathways that enhance drug resistance or adapt to host nutritional constraints. Energy deprivation likely causes GBS to “optimize” away the energy expenditure required for virulence production, prioritizing its own survival [62]. Based on our invasion assay results and integrated metabolomic and transcriptomic analyses, we hypothesize that, under baicalein stress, GBS prioritizes energy allocation for survival, leading to reduced transcription of most virulence genes and diminished invasive capacity. Baicalein not only inhibits GBS but also activates the Nrf2-HO-1 pathway, which reduces oxidative stress and inflammation in bMECs and promotes cell survival.

## 5. Conclusions

Baicalein inhibited the invasion and adhesion of GBS HB31. Metabolomics results showed that baicalein downregulated key amino acids and nucleotide metabolites, induced an increase in the NADH/NAD^+^ ratio and a decrease in ATP levels, thereby suppressing energy metabolism. Transcriptomic results further revealed that baicalein interfered with quorum sensing, carbon metabolism, two-component systems, and oxidative phosphorylation pathways in GBS. Based on the findings, it is speculated that baicalein inhibits energy production in GBS, leading to energy reallocation that prioritizes bacterial survival, which in turn reduces its invasive capacity. At non-bactericidal concentrations, baicalein also alleviates oxidative stress and the inflammatory response in bMECs. This study provides a basis for the development of baicalein as an anti-virulence agent.

## Figures and Tables

**Table 1:** Primers of Virulence genes of GBS.

Gene Name	Sequence (5′–3′)
*16sRNA*	F: TCGTGTCGTGAGATGTTGGG
	R: GTTTGTCACCGGCAGTCAAC
*cylE*	F: GGAAGTTACCCGATTGAGCA
	R: TGCCAGGAGGAGAATAGGAA
*covS*	F: GATCCTGCAGAAACAATGATGTTTTGCCTCA
	R: GACTCGAGAAAAGAGCTCGTTGATCAGG
*cfb*	F: ATGAACGTTAAACATATGATGTATCTATCT
	R: GATTGAGCAGCATTCAAACAGCATTAAATAA
*hylB*	F: ATGAAGCAAAGAAACTATCATCTTCTGTTTGATCTA
	R: TACTTTAAGCAGCTATCTTTTAAGGCCTTATCATAG
*fbsB*	F: ATCAGCAGCGATTCTATCGCTAGCAGTAACAGTA
	R: GAAGTTACTGAGATGAGTAAATCAATCTCATC

**Table 2:** Primer sequences for antioxidant and inflammation-related genes of bMECs.

Gene Name	Sequence (5′–3′)	Accession Number
*β-actin*	F: CCTCACGGAACGTGGTTACA R: TCCTTGATGTCACGCACAATTT	NM_173979.3
*SOD*	F: ATCCACTTCGAGGCAAAGG R: ACGTGGAATCCATGATCACC	NM_174615.2
*Nrf2*	F: CCAGCACAACACATACCA R: TAGCCGAAGAAACCTCATT	NM_001011678.2
*GSH-Px*	F: CTTCAACCTGTCCTCCCT R: GGTCATTCATCTGGGTGT	NM_174076.3
*CAT*	F: TCACTCAGGTGCGGACTTTC R: TGGATGCGGGAGCCATATTC	NM_001035386.2
*HO-1*	F: GGCAGCAAGGTGCAAGA R: GAAGGAAGCCAGCCAAGAG	NM_001014912.1
*TLR4*	F: CAGGGCAGGGAAAGTCAA R: AGGAAAAGTGAGCCAAGACC	NM_174198.6
*NF-κB*	F: TCACCAGGGAAGGATCTACG R: AGCGGCTCAACAGGTACAGT	NM_001080242.2
*IL-6*	F: CAAGCGCCTTCACTCCATTC R: GATTTTGTCGAC CATGCGCT	NM_173923.2

**Table 3:** Changes in selected genes and metabolites of GBS under the action of baicalein.

Gene/Metabolite Names	Log_2_FC	Baicalein
*hylB*	−0.50548	normal
*cylE*	1.389814	up
*scpB*	1.645634	up
*fbsA*	0.188745	normal
*cfb*	−0.50818	normal
*pbsP*	−0.80702	down
*recj*	1.215873522	up
*tpx*	0.665061	up
*phoB*	−0.27819	normal
*phoR*	0.627724	up
*walR*	0.666864	up
*walK*	0.650431	up
*saeR*	−0.22843	normal
*saeS*	1.438536	up
*luxS*	1.003964	up
*tet(O)*	1.59807	up
*ermB*	−0.13817	normal
*vanY*	0.642241	up
Phosphoenol pyruvate	−5.515789825	down
Phosphoric acid	0.695349	normal
NAD^+^	−3.523905473	down
NADH	13.65954895	up
ATP	−41.6059333	down
Acetoin	2.114254023	up

## Data Availability

The original contributions presented in this study are included in the article. Further inquiries can be directed to the corresponding authors.
